# Moving Your Sons to Safety: Galls Containing Male Fig Wasps Expand into the Centre of Figs, Away From Enemies

**DOI:** 10.1371/journal.pone.0030833

**Published:** 2012-01-25

**Authors:** Hui Yu, Stephen G. Compton

**Affiliations:** 1 Key Laboratory of Plant Resources Conservation and Sustainable Utilization, South China Botanical Garden, the Chinese Academy of Sciences, Guangzhou, China; 2 School of Biology, Faculty of Biological Sciences, University of Leeds, Leeds, West Yorkshire, United Kingdom; University of Northampton, United Kingdom

## Abstract

Figs are the inflorescences of fig trees (*Ficus* spp., Moraceae). They are shaped like a hollow ball, lined on their inner surface by numerous tiny female flowers. Pollination is carried out by host-specific fig wasps (Agaonidae). Female pollinators enter the figs through a narrow entrance gate and once inside can walk around on a platform generated by the stigmas of the flowers. They lay their eggs into the ovules, via the stigmas and styles, and also gall the flowers, causing the ovules to expand and their pedicels to elongate. A single pollinator larva develops in each galled ovule. Numerous species of non-pollinating fig wasps (NPFW, belonging to other families of Chalcidoidea) also make use of galled ovules in the figs. Some initiate galls, others make use of pollinator-generated galls, killing pollinator larvae. Most NPFW oviposit from the outside of figs, making peripherally-located pollinator larvae more prone to attack. Style length variation is high among monoecious *Ficus* spp. and pollinators mainly oviposit into more centrally-located ovules, with shorter styles. Style length variation is lower in male (wasp-producing) figs of dioecious *Ficus* spp., making ovules equally vulnerable to attack by NPFW at the time that pollinators oviposit.

We recorded the spatial distributions of galled ovules in mature male figs of the dioecious *Ficus hirta* in Southern China. The galls contained pollinators and three NPFW that kill them. Pollinators were concentrated in galls located towards the centre of the figs, NPFW towards the periphery. Due to greater pedicel elongation by male galls, male pollinators became located in more central galls than their females, and so were less likely to be attacked. This helps ensure that sufficient males survive, despite strongly female-biased sex ratios, and may be a consequence of the pollinator females laying mostly male eggs at the start of oviposition sequences.

## Introduction

The mutualism between fig trees (*Ficus* spp.) and their fig wasp pollinators (Agaonidae) is one of the most intensively studied of plant-insect interactions [1], yet fig wasps are rarely mentioned in general discussions of gall-forming insects [2]–[4], (but see [5]). This reflects the small size of fig wasp galls and their internal location, which means that the galls are not visible externally. Fig wasp galls and many of the other unusual features of fig wasp biology are dictated by the structure of *Ficus* inflorescences (figs, also known as syconia), inside which adult females lay their eggs and immature pollinators develop. A fig is formed like a hollow ball, lined on the inside by hundreds or thousands of tiny female flowers, each of which can produce one fig wasp or one seed. Gall induction coincides with oviposition and pollination and takes place after the entry into a fig of one or more foundress pollinator fig wasps. Galling results in very rapid growth of the ovule and elongation of its supporting pedicel [6], [7] and provides sufficient resources for a single fig wasp larva to develop. The galled ovules generally remain discrete and do not fuse with each other. Along with local changes to individual flowers, galling also inhibits abortion of the whole fig (stimulative parthenocarpy *sensu* [8]) though pollination can also be required [9].

Parasitoid and inquiline non-pollinating fig wasps (NPFW) belonging to several groups of chalcid wasps (Chalcidoidea) are a major source of mortalities among pollinator fig wasps [10]. Inquilines and parasitoids are distinguished by their larval feeding behavior, with inquilines feeding on plant tissue as well as destroying the larvae of other fig wasps, but both groups always result in the deaths of pollinator larvae within shared galls. Most NPFW lay their eggs from the outside of the figs, resulting in more centrally located galls being more difficult to find or reach and consequently more central galls often benefitting from reduced levels of attack [11]–[15]. Oviposition by the NPFW is made possible by their elongate ovipositors, which in some species are longer than the rest of their bodies. The partial refuge (‘enemy-free space’) offered to more centrally located pollinator larvae is similar to that recorded for some other gall-forming insects, where larger gall diameters can result in reduced parasitism [16]–[21] and to other gregarious endophytic insects whose larvae develop at varying distances from the surface [22].

Fig wasps have a haplo-diploid sex determination mechanism, with males and females developing from unfertilised and fertilised eggs respectively. The sex ratios of pollinator fig wasps are strongly female biased, but the extent of this bias often varies according to how many foundress females share a fig. As the number of foundresses increases, typically so does the proportion of male offspring produced [23]–[27]. These changes are broadly in line with optimality predictions based on the extent of local mate competition experienced in the fig, and levels of inbreeding [28]. Most sex ratio models assume that one surviving male is sufficient to fertilize all females in the brood [29], [30] and optimal sex allocation strategies can lead to appreciable levels of virgin females [30], [31]. Because foundresses lay relatively few male eggs, there is a danger that even moderately high mortality levels will result in some figs containing no male offspring [32]. This means not only that no matings will occur (females are mated while still in their galls), but also that all the females are likely to die inside their fig, because they depend on the males to chew an exit hole through the fig wall. Consequently, the risk of mortalities among rare males leads to ovipositing females facing a trade-off between minimizing the number of sons produced (to avoid superfluous males), and insuring against the possibility that all the sons in a patch will die, leaving numerous daughters that are also likely to die without reproducing [33]. The result is that extra (‘insurance’) male eggs may be required to ensure that some survive [29], [34].

At the time when foundresses enter the figs there is a distinct central cavity inside which they can move around, pollinate and probe the styles with their ovipositors. The central cavity is lined by the stigmas, which typically form a uniform surface, the synstigma. *Ficus* species have either monoecious or functionally dioecious breeding systems [35], [36]. In monoecious species, each fig produces a mixture of wasps, seeds and pollen. Because the ovaries in monoecious figs are situated at varying distances from the central cavity the synstigma is achieved by the flowers having styles and basal pedicels of varying lengths (flowers with longer styles have shorter pedicels). Longer-styled flowers are more likely to produce seeds and shorter-styled flowers are more likely to produce pollinator offspring [37], [38]. The preference amongst foundresses for shorter-styled flowers means that most of their eggs tend to be laid in ovules that are initially more central, perhaps in response to selection to avoid NPFW, though other factors may also be important [12], [39]–[41].

**Figure 1:**
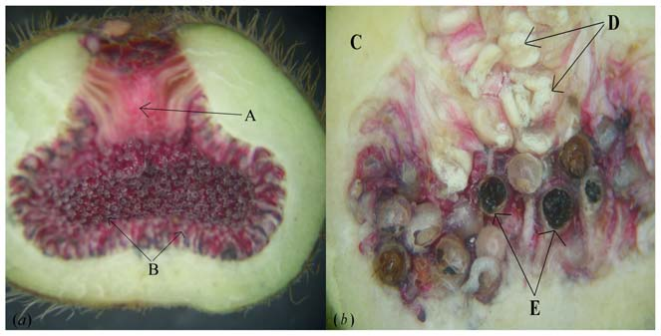
The internal organization of male figs of *F. hirta* at the stages when they are entered by foundress fig wasps and when the next generation of fig wasps are almost ready to emerge. (*a*). B phase male fig of *F. hirta* showing (A) the ostiole through while *Valisia javana* females enter the fig to oviposit and (B) the small female flowers, with short styles, through which the fig wasp lays her eggs. Male flowers are tiny at this time. (*b*). Late C phase male fig of *F. hirta* showing (C) the fig wall, (D) mature male flowers clustered at the base of the ostiole and (E) galled ovules containing fig wasp pupae.

In dioecious *Ficus* species the male trees produce ‘male’ figs that contain male flowers and female flowers with short styles. They produce no seeds, but act as nurseries for developing pollinator larvae. Female trees produce ‘female’ figs that contain only female flowers and they only produce seeds. This is achieved by having flowers with much longer styles, which prevent foundress females from ovipositing, though they still pollinate the flowers [26]. Style length variation in male figs is much less than that found in monoecious figs of similar size, resulting in the position of their associated ovules relative to the outside of the fig also varying little at the time when pollinators enter [42], (figure 1*a*). After pollination and oviposition the ovules enlarge and their pedicels elongate to fill all the available space within the fig (figure 1*b*), which is also increasing in overall size. The central cavity re-appears later, when the fig diameter expands in time for the next generation of wasps to emerge from their galls, mate and then vacate the figs.

We recorded the spatial distribution of galled ovules in figs of the dioecious fig tree *F. hirta* Vahl at the stage when the fig wasps had completed their development and would soon emerge, noting the position of the galls relative to the periphery of the figs, and the species and sex of the fig wasps they contained. The spatial distributions of galled ovules were also compared with those of ovules that had not been galled and with ovules in younger figs before galling had been initiated. The following questions were addressed: Do NPFW have the same distribution patterns as pollinator fig wasps? Are male and female pollinators distributed at similar distances from the periphery, are they equally likely to be attacked by NPFW and if not, do NPFW alter the realized sex ratios of the pollinators? We then compared the extent of variation in the positions of mature galls with the initial positions of the ovules, prior to galling, to determine how the observed spatial patterns in mortalities are generated.

## Materials and Methods

### Study site and species

Our studies were carried out at the South China Botanical Garden (SCBG), in Guangdong Province (113°11′E, 23°11′N). The area has a subtropical maritime climate, with an annual mean temperature of almost 22°C. The dry season extends from October to March, the wet season from April to September. No specific permissions were required for these locations/activities. Because the location is not privately-owned or protected in any way, and field studies did not involve endangered or protected species.

*Ficus hirta* Vahl is a dioecious shrub or small tree that produces roughly spherical figs on its branches that reach up to 3 cm in diameter at maturity [43]. Like other dioecious fig species, female trees bear female figs that contain only female flowers and produce seeds. Male trees produce functionally male figs that contain both male and female flowers. Female figs contain an average of around 850 female flowers, and male figs around 800 (exceptionally >1000) female and 100 male flowers [44]. Male figs develop asynchronously on individual trees, with production of receptive and mature male figs peaking at the same time of year [45]. Crop sizes are small, with often just one wasp-releasing fig present at any one time.

As with other *Ficus* species, the development of *F. hirta* figs is protogynous and can be divided into the following phases (modified from Galil and Eisikowitch, 1968 [46]): (A) young immature figs; (B) figs where the ostiole is temporarily open and fig wasps can enter to lay their eggs and pollinate the female flowers; (C) the longest phase, where fig wasp larvae and seeds are developing. Each wasp larva feeds on the contents of a single ovule in male figs, and one seed develops per ovule in female figs; (D) adult wasps of the next generation (in male figs) mate while the females are still in their galls. Females then emerge from their galls and in passively-pollinated species such as *F. hirta* become covered in pollen that is released from the mature male flowers, before escaping in search of phase B figs. Female figs have no equivalent phase because no male flowers and wasp offspring are present. (E) Finally, male figs shrivel and eventually fall to the ground, whereas female figs become soft and fleshy, offering a food reward to avian seed dispersers.

The pollinator of *F. hirta* is recorded as *Blastophaga javana* Mayr (Agaonidae; Wiebes 1993 [47]. Its generic placement has recently been revised and the species is now known as *Valisia javana* (Mayr) [48]) Molecular studies (H. Yu, unpublished) also indicate that two very similar species pollinate this tree in China, though only one species occurs at SCBG). The number of adult females entering each fig (foundresses) at SCBG averages 1.7 and ranges from 1–9 [44]. Three species of non-pollinator fig wasps also utilize male figs of *F. hirta* at SCBG: *Philotrypesis josephi* Balakrishnan, *Sycoscapter hirticola* Balakrishnan and *Sycoscapter simplex* Mayr [49]. No species that independently gall the ovules (other than the pollinator) were present. Females of these non-pollinators oviposit from the outside of the figs, using their long ovipositors to reach the ovules where their larvae develop. The NPFW females lay their eggs into C phase figs, into galled ovules where fig wasp larvae are present *S. simplex*, which was rare at the study site, has a noticeably longer ovipositor than the other two species, suggesting that it oviposits into older figs than the others [12]. Only a single adult pollinator or NPFW emerges from each gall. Adult females of the next generation emerge through exit holes produced through the ostiole.

### Style and pedicel lengths in male figs

Variation of style length (the distance from where the style joins the ovary to the top of the stigma) was measured in 248 flowers from 6 male figs collected at the stage when they are pollinated (Phase B). Sections of the figs were removed at random and all the flowers present in the sections were measured. The styles were measured to the nearest 0.1 mm using an eyepiece graticule mounted to a dissecting microscope. Pedicel lengths of 212 flowers from 11 figs at phase B were measured in the same way. During Phase B the tops of the stigmas are aligned to form a platform that delimits a central lumen from where the fig wasp oviposits. Flowers with longer styles therefore have shorter pedicels and have ovules that are closer to the periphery of the fig.

### The impact of non-pollinators on pollinator numbers and sex ratios

Monthly collections at SCBG between July 2002 and June 2003 (from more than 20 trees) produced a total of 107 late C phase male figs. Often trees had only a single such fig at any one time, precluding partitioning of within and between crop effects. The figs were placed individually in separate mesh-covered containers to allow adult fig wasps to emerge. The figs were then searched for any remaining wasps, and their total numbers and sexes recorded. Non-pollinator species were not counted separately because males could not be assigned to species.

### The location of fig wasp galls within male figs

During B phase, style lengths of individual flowers are negatively correlated with their pedicel lengths, but variable growth of the pedicels of galled flowers results in this relationship becoming less clear as figs mature. In *F. hirta* this results in style lengths being a poor measure of the location of developing fig wasp larvae relative to the periphery of the figs (figures 1*a and 1b*). We therefore recorded the spatial distribution of galled ovaries during late C phase by measuring the distance from the inside of the fig wall to the innermost point of each ovule. At this stage the galls contained pupae and adult fig wasps. The location and contents of 792 galled ovules were selected at random as before from 26 male figs collected from fifteen trees between September and December 2005 and in June 2006. Males and females of pollinators and NPFW were distinguished. Galled ovule location was measured as the distance from the inside of the fig wall to the inner edge of each ovule. This distance includes the length of both the pedicel and the ovule. Pedicel length and ovule size were recorded separately for 429 of the flowers (obtained from twelve figs). Measurements were made to the nearest 0.1 mm. The internal diameters (between the inner edges of the fig walls, at right angles to the ostiolar axis) of 26 late C phase figs were also measured.

### Data analysis

All tests were carried out using SPSS 11.0 (SPSS Inc., Chicago, IL, USA). Spearman rank correlations examined the relationship between ovule size and pedicel length. Logistic regressions examined the relationship between pedicel lengths and the presence of male pollinators, female pollinators or NPFW in their associated ovules. One-way analysis of variance (ANOVA) assessed the relationships between pollinator numbers and sex ratio (proportion of males, arc sign square root transformed) and compared pollinator abundance in figs with or without NPFW. The locations of galls containing male and female pollinators and NPFW were also compared using ANOVA. Contributions to significant ANOVA effects were examined using Tukey *post hoc* tests. Spearman rank correlations assessed relationships between pollinator abundance, pollinator sex ratios, and the numbers of NPFW.

## Results

### Style and pedicel lengths in male figs

**Figure 2:**
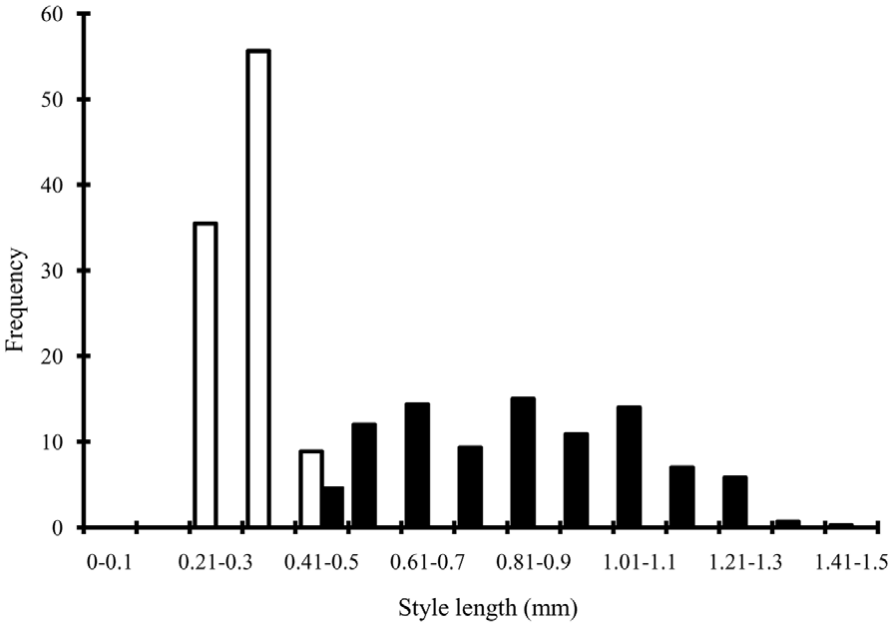
Style length variation in B phase male figs of *F. hirta*, compared with the much greater variation in style lengths exhibited during B phase in a monoecious *Ficus* species (*F. burtt-davyi*).

Styles length variation in B phase male figs (the stage when pollinators enter to lay their eggs) was unimodal, with a range of 0.24 mm between the longest and shortest styles (figure 2). This range in style lengths is much smaller than that seen at B phase in monoecious figs, an example of which is also provided in figure 2 (with B phase figs of the southern African *Ficus burtt-davyi* [37].

Pedicel lengths in phase B phase figs of *F. hirta* were much shorter than at the end of C phase, when pedicel growth had ended and the wasps had completed their development (Mean ± SE at B phase = 0.33±0.13 mm, n = 212, compared with 2.01±1.12 mm, n = 502; F [1] = 477.0, P<0.001).

### The impact of NPFW on pollinator numbers and sex ratios

**Figure 3:**
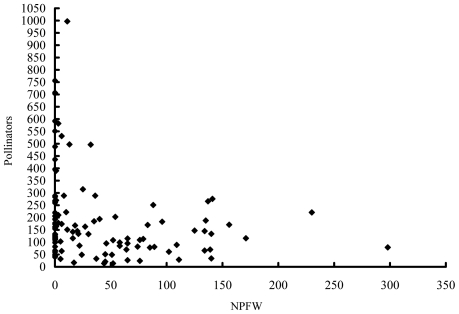
The relationship between pollinator and NPFW numbers in figs of *F. hirta.*

*V. javana* and three species of NPFW were present in the 107 *F. hirta* figs where all the fig wasps were recorded. Fig wasps numbers varied greatly between figs, but averaged about 230 (table 1). Pollinators were present in all but two of the figs (where all are presumed to have been killed by the NPFW), NPFW were present in 68% (table 1). Pollinator numbers were highly variable, even in figs with no NPFW, but negatively correlated with NPFW (Spearman rank correlation = −0.317, P<0.001, n = 107) (figure 3).

**Table 1:** The fig wasps in 107 *Ficus hirta* figs at SCBG.

		Fig occupancy (%)	Mean	SD	Range
Pollinators	Female	105 (98.0)	145.59	148.85	0–852
	Male	103 (96.0)	44.25	59.84	0–294
	Total	105 (98.0)	189.84	178.81	0–997
NPFW	Total	73 (68.0)	41.93	55.66	0–298
All fig wasps	Total	107 (100)	231.78	172.97	22–1008

**Figure 4:**
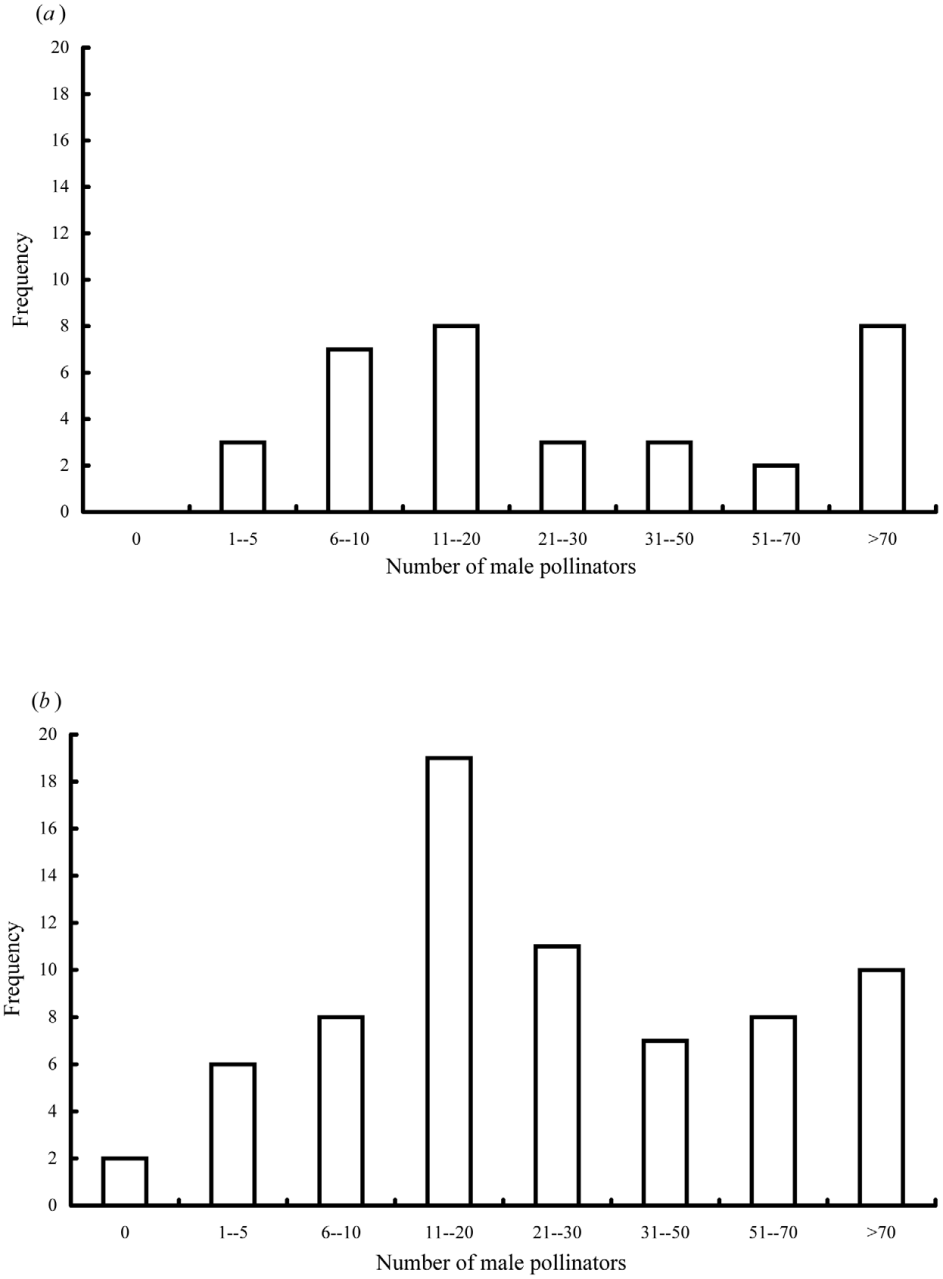
The numbers of male V. *javana* present in figs of *F. hirta*. (*a*) Figs with NPFW absent (n = 34 figs) or (*b*) present (n = 71 figs).

Pollinators represented 82% of the total wasps reared from the figs, suggesting an 18%, mortality rate due to NPFW if each adult NPFW had developed at the expense of one pollinator. The 34 figs that contained only pollinators suggest this is an underestimate of the true impact of the NPFW. Total pollinator numbers were 36% lower in the figs that were shared with NPFW, compared with figs where NPFW were absent (table 2). A combination of damage inflicted during NPFW ovipositor probing and an increased likelihood of early mortality in galls that had NPFW eggs laid in them is the likely explanation for the reduced numbers of pollinators emerging from the figs. If pollinator numbers per fig before the impact of NPFW had been the same, then for each adult NPFW, two pollinators had been killed. Only two of the 105 figs occupied by *V. javana* did not have males present. Both were figs shared with NPFW (figure 4). Figs that contained more male than female pollinators had been entered by one or more virgin foundresses, which can only produce male offspring.

**Table 2:** *Valisia javana* abundance and sex ratios (proportion males) in figs with and without NPFW (two figs without *V. javana* are excluded).

	NPFW absent(n = 34 figs)		NPFW present(n = 71 figs)	
	Mean	SD	Mean	SD
Pollinator females	200.76	170.25	119.89	131.25
Pollinator males	51.15	62.77	41.04	58.59
Total pollinators	251.91	205.51	160.93	158.26
Total fig wasps	251.91	205.51	222.40	156.24
Pollinator sex ratios	0.21	0.20	0.27	0.24

**Figure 5:**
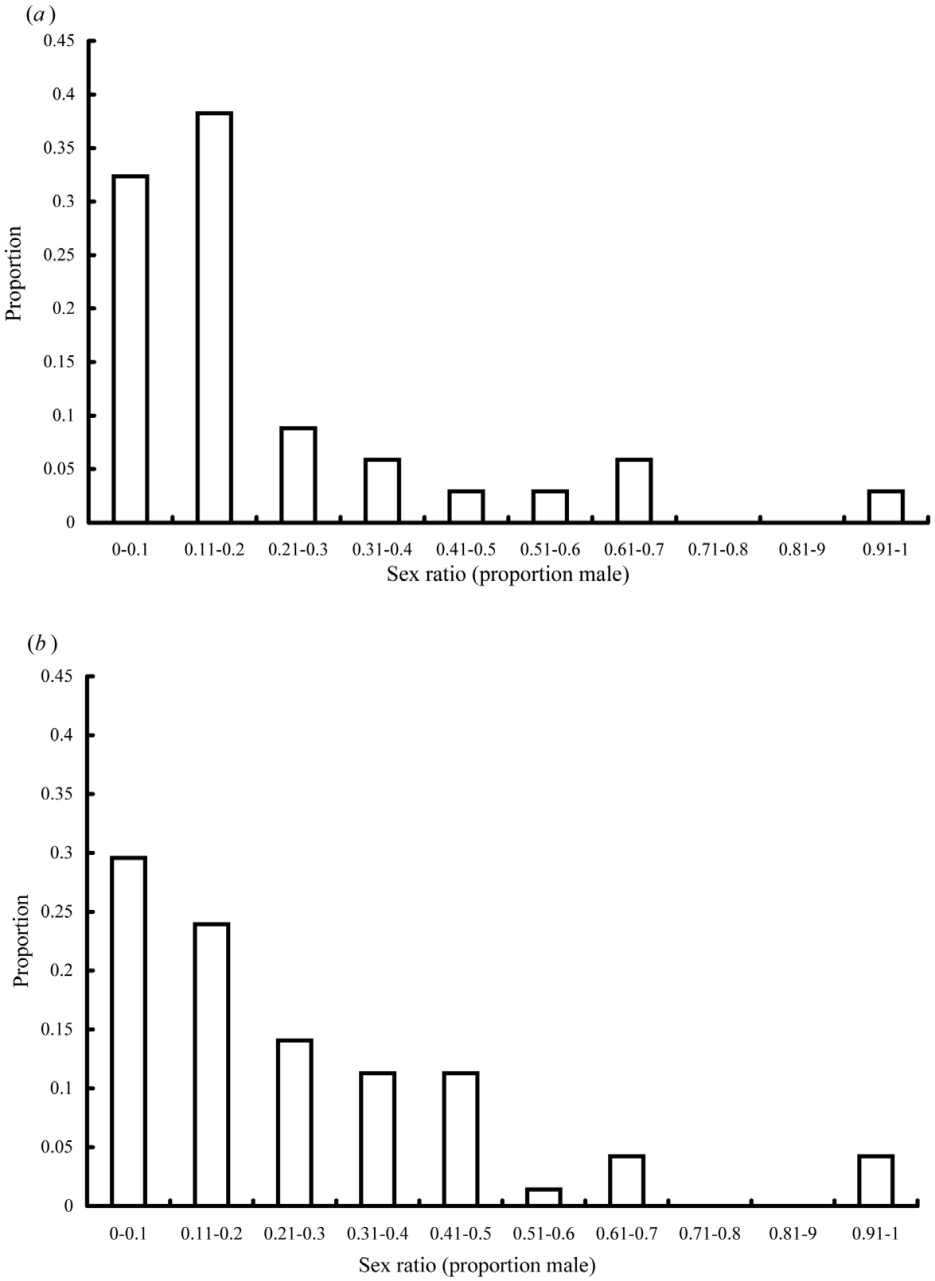
Variation in *V. javana* sex ratios (proportion males). (*a*) 34 figs where NPFW were absent and (*b*) 71 figs where NPFW were present.

Pollinator sex ratios (proportion of males) varied greatly between figs (figure 5; n = 20,313 fig wasps from 105 figs; mean sex ratio = 0.25, SD = 23.1). Overall pollinator sex ratios were 0.27 in figs shared with NPFW, and 0.21 in figs where pollinators were present alone, but as sex ratios were often highly variable within groups this difference was not significant (arc sign transformed sex ratios, F = 1.830, P = 0.179). Figs lacking NPFW contained significantly more female and total pollinators than figs with NPFW, but the numbers of males did not differ (table 2; ANOVA, total pollinators, F = 5.629, P = 0.02, female pollinators F = 6.562, P = 0.012, male pollinators F = 0.518, P = 0.478).

Numbers of female and total pollinators were negatively correlated with NPFW abundance in the figs, but not males or sex ratios (table 3). The same result was obtained when only figs with sex ratios of 0.4 or more (that were presumed to have received un-mated foundresses) were excluded (table 3). Sex ratios were also not significantly related to parasitism rates (proportion of all fig wasps that were NPFW): Spearman rank correlation, r = 0.159; P = 0.106; N = 105. This was also the case when figs with more than 40% males were excluded (r = 0.094; P = 0.389; N = 86).

**Table 3:** The relationship between NPFW abundance and numbers and sex ratios of pollinators in 107 figs of *F. hirta* (Spearman rank correlations).

	All figs		Sex ratio ≤0.4(n = 86 figs)	
	r	P	r	P
Females	−0.288	0.003	−0.267	0.013
Males	−0.120	0.217	−0.148	0.175
Total pollinators	−0.317	<0.001	−0.255	0.018
Sex ratios	−0.036	0.713	0.120	0.270

Figs with pollinator sex ratios of less than 0.4 were examined separately because they were less likely to have been entered by un-mated foundresses. N = 105 for (arc sign square root transformed) sex ratio comparisons.

### Gall sizes, pedicel lengths and the location of fig wasp galls within figs

Galled ovules at the end of phase C that contained male and female pollinators and NPFW were all about one mm in length and did not differ significantly in size (ANOVA, F [3] = 1.684, P = 0.17, N = 429 ovaries, table 4). Ovule size and pedicel length were not related (Spearson rank correlation r = 0.02, P = 0.685, n = 429). Pedicel lengths were longer in flowers that had been galled than others that had not (table 4). Pedicel lengths of ovules occupied by pollinators varied between 0.1 mm and 6.17 mm, and for NPFW between zero and 4.35 mm. One way ANOVA followed by Tukey *post hoc* tests showed that ovules occupied by pollinators and NPFW had significantly different pedicel lengths (F [3] = 41.606, P<0.001). Pedicel lengths also varied significantly between ovules containing male and female pollinators (P<0.001) and between both pollinator sexes and NPFW (both P<0.01). The pedicel lengths of ovules with male and female NPFW did not vary significantly (P = 0.909). Male pollinators occupied ovules with the longest pedicels, NPFW occupied the ovules with the shortest.

**Table 4:** The size of late C phase ovules containing *F. hirta* pollinators or NPFW and the lengths of their pedicels compared with those of flowers that had not been galled.

		Gall size (mm)		Pedicel length (mm)	
	N (ovaries)	Mean	SD	Mean	SD
Female pollinators	117	1.08	0.17	2.15	1.02
Male pollinators	130	0.96	0.6	2.86	1.12
Female NPFW	115	1.06	0.15	1.52	0.91
Male NPFW	67	1.03	0.15	1.63	0.96
Ungalled flowers	73	-	-	1.40	0.64

**Figure 6:**
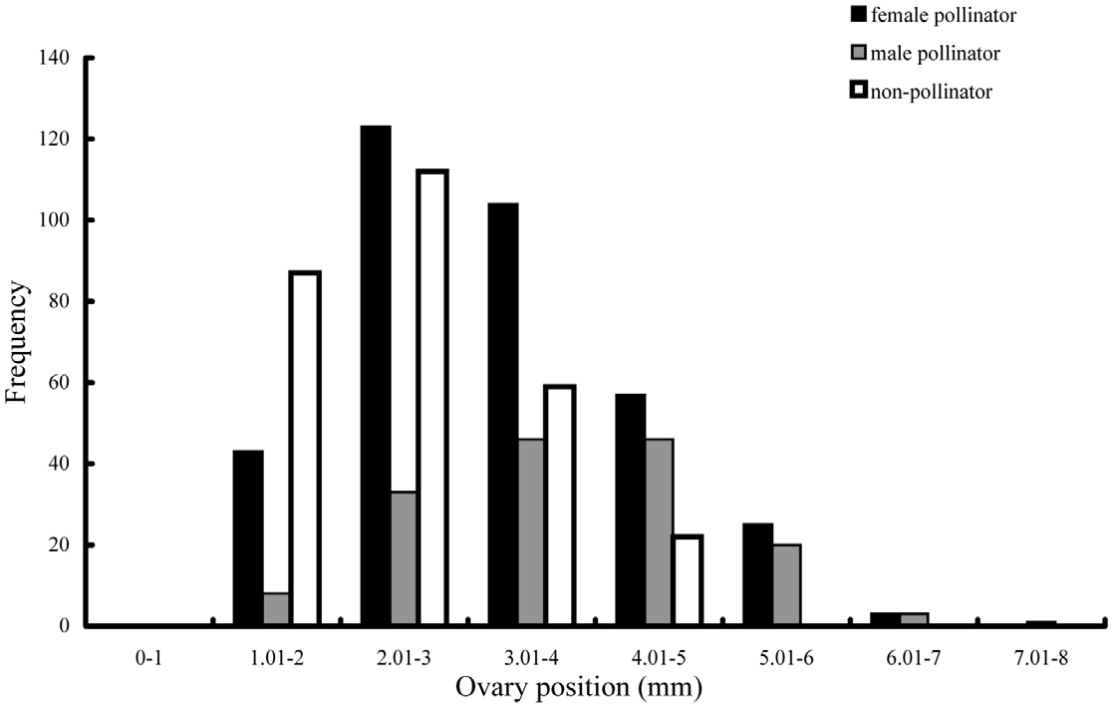
The contents of galled ovules in relation to their position within *F. hirta* figs. Longer ovary positions were closer to the centre of the figs.

The internal diameter of the male figs at the end of C phase was 10.1±1.53 mm (Mean ± SD, n = 26 figs). As the lengths of the roughly spherical ovules containing wasps were around 1 mm (table 4), this would be sufficient to accommodate more than four concentric layers within the figs, but their arrangement was much more haphazard (figure 1*b*). The inner edge of the ovules at late C phase was always at least one mm from the fig wall, because the ovules themselves were about one mm long. The space available declined towards the centre of the figs, so fewer ovules were located there, but there were also relatively few sessile ovules (with no measurable pedicel) located next to the fig wall (figure 6).

Ovule positions indicate the relative distances that a NPFW female would have to probe to reach the inner edge of that ovule, after its ovipositor had first penetrated the fig wall. Their absolute values will be greater than those experienced by the ovipositing NPFWs, because the measurements were taken at the end of C phase, after oviposition was completed, and the figs had subsequently grown in size. In total, the positions of 792 fig wasps were recorded (table 5). Ovules containing different species varied significantly in position (F [3] = 48.967, P = <0.001, n = 792; figure 6). One way ANOVA followed by Tukey *post hoc* tests showed that ovules occupied by different species tended to be located in different positions. Positions varied significantly between ovules containing male and female pollinators (P<0.001) and between both pollinator sexes and NPFW (all P<0.001). There was no difference in the positions of ovules containing male and female NPFW (P = 0.998). Recorded patterns of occupancy based on ovule position therefore closely reflected variation in pedicel lengths.

**Table 5:** The positions of ovules containing pollinators or NPFW in *F. hirta* figs.

		Ovule position (mm)	
	N (ovules)	Mean	SD
Female pollinator	355	3.28	1.09
Male pollinator	157	3.82	1.12
Female NPFW	183	2.60	0.90
Male NPFW	97	2.62	0.94

Around 50% of the fig wasps in the more peripheral ovules of the two shortest length classes were NPFW, compared with less than 30% in the more central ovules (table 6). No NPFW were recorded from the small number of most central ovules. Pollinator sex ratios also became progressively less female biased towards the centre of the figs (table 6). Logistic regressions confirmed that ovules with shorter pedicel lengths were more likely to contain NPFW than pollinators (B = −0.896, Wald = 63.157, P<0.001) and female rather than male pollinators (B = −0.431, Wald = 23.647, P = <0.001, n = 512).

**Table 6:** The contents of ovules in relation to their position in *F. hirta* figs.

	Pollinators			NPFW		
Ovary position (mm)	Female	Male	Sex ratio(Proportion male)	Female	Male	% of total fig wasps
1–2	43	8	0.157	56	31	0.63
2–3	123	33	0.212	73	39	0.42
3–4	104	46	0.307	40	19	0.28
4–5	57	46	0.447	12	7	0.16
5–6	25	20	0.444	2	1	0.06
6–7	3	3	0.50	0	0	0
7–8	0	1	1.0	0	0	0

## Discussion

Galled ovules that contained male pollinator fig wasps were concentrated towards the centre of *F. hirta* figs, where they were less likely to be subject to attack by NPFW. The concentration of NPFW towards the periphery of *F. hispida* figs reflects the greater accessibility of more peripheral pollinator galls to female NPFW ovipositing from the outside of the figs. In monoecious figs there is considerable variation in pedicel and style lengths at the time when pollinators induce the galls, leading to ovules already being located at varying distances from the periphery and raising the possibility that they may vary in their suitability for galling [50]. The much smaller variation in the placement of the ovules in *F. hispida* and other dioecious figs at the time they are galled by the pollinators means that the differences seen in their eventual locations are generated after galling takes place. Male larvae benefitted from developing in galled ovules that displayed greater average pedicel growth than those of females, positioning them towards the centre of the figs where they were less likely to be attacked. Male pollinators that develop in more central galls may also benefit in other ways, as they can emerge more quickly into the central lumen and gain easier access to females [39], [51], [52].

Price *et al.* [53] have argued that improved nutrition and protection from physiological stresses are the major benefits that have driven the evolution of gall production in insects, rather than protection from natural enemies. The rich parasitoid faunas often associated with gall-forming insects provide support for their conclusion, as they often generate high mortality rates [54]. The nutritive benefits of galling by pollinator fig wasps are clear cut, as larvae only develop in galled ovules, but the extent of gall development is also significant for *V. javana*, because the limited space available generates competition for the partial refuge from NPFW afforded by ovules in more central locations.

Pollinator numbers were reduced by around one third in *F. hirta* figs where NPFW were present, a rate of loss that is found in many *Ficus* secies [55]–[57]. NPFW can reduce the numbers of pollinator offspring by destroying pollinator larvae (parasitoids or inquilines) or be independent gall-forming NPFW that compete with pollinators for oviposition sites [58]. The timing of oviposition by the *Sycoscapter* and *Philotrypesis* NPFW associated with *F. hirta* is weeks after the pollinators lay their eggs, so their impact resulted from the destruction of pollinator offspring. The declines in the numbers of pollinator offspring in figs shared with NPFW were not matched by equivalent numbers of adult NPFW, suggesting that ovipositor probing by the NPFW females may also kill many developing pollinator larvae.

Pollinator figs wasps typically produce highly female-biased broods, unless they have remained virgins (constrained females *sensu* [31], [59]). In figs where parasitism rates are high, and the sexes are equally lively to be attacked by NPFW, there is a danger that all the male pollinators will be killed. This has a disproportionate effect, because in addition to failing to mate, the female pollinators will not be able to emerge from these figs, unless NPFW males are able to chew an exit hole, which males of most *Philotrypesis* and *Sycoscapter* species cannot achieve [57]. In response to this eventuality, pollinator fig wasps may produce more male offspring than would otherwise be optimal in order to provide ‘insurance’ against the destruction of all the males in a fig [34].

Most NPFW females lay their eggs while standing on the outer surface of a fig, using their extremely long ovipositors [60]. This means that pollinator larvae developing closer to the fig surface are often likely to be encountered first by probing NPFW females and are often subject to greater levels of parasitism than more centrally located larvae [15]. Mean style lengths in monoecious figs are relatively long and there is a large variance in style lengths, whereas style lengths in male dioecious figs are much shorter and variance is small [42]. In monoecious figs this results in style lengths being indicative of the subsequent location of the ovules, and the degree of their subsequent exposure to NPFW. In dioecious figs such as *F. hirta*, the location of developing larvae is largely determined by the degree of growth of the pedicels that support the galled ovaries, rather than the style length of the flower. The range in style lengths when the eggs are laid is very small, much less than one mm, whereas by the time the wasps are ready to emerge from their galls, the range in their positions is about 7 mm. Reflecting this, the variance ratio for style lengths in B phase male *F. hirta* figs is 0.005 (mean = 0.35 mm, n = 48), whereas by the time that pollinators have completed their development the variance ratio for pedicel lengths (style lengths can no longer be measured then because of decay) climbs to 0.76 (mean = 2.11 mm, n = 429). The more central location of male *V. javana* offspring at this time could result from greater pedicel growth by those flowers with the shortest styles (assuming that male offspring are preferentially located in such flowers, as is often the case in monoecious figs [12], or independently of style length, galls containing males may be stimulated into greater petiole elongation than those that contain females.

The extent to which gall development in fig wasps is controlled by ovipositing females or their larvae is unclear, but galled ovules expand very rapidly after oviposition, implicating the liquid injected by ovipositing females as a galling agent [7]. Some pollinators lay most of their male eggs early in an oviposition sequence [26], [61] and if this is true of *V. javana* then it may be that ovipositing females release more of their galling stimulants with their first eggs. Alternatively, if most male eggs are laid first, then they may simply get a head start in terms of competition for the resources needed to expand their petioles and occupy the limited central space. The same arguments apply to the first foundresses that enter a fig and it may be their male offspring, rather than those of foundresses that enter later, that occupy the most central positions [62]. However it is achieved, the effect of differential growth of galls containing males is to place them in a partial refuge, where the chance of being killed by NPFW is much reduced. Consequently, there is less need for ‘insurance’ males than would otherwise be the case to ensure that each fig has at least one surviving male offspring [32]. Among the 105 *F. hirta* figs that contained *V. javana* only two lacked males, despite estimated losses of 32% due to NPFW. A similar concentration of male pollinator offspring towards the centre of figs has also been recorded in another dioecious species, *F. hispida* [53], suggesting that galls containing male fig wasps may often display greater pedicel growth than those containing females.
